# Automated deep learning detection of orthodontically induced external apical root resorption in maxillary incisors on panoramic radiographs

**DOI:** 10.1186/s40510-026-00610-9

**Published:** 2026-02-26

**Authors:** Samet Özden, Betül Kula, Mahmut Tankuş

**Affiliations:** 1 İnönü University, Malatya, Turkey; 2 İstanbul Galata University, İstanbul, Turkey; 3 Harran University, Şanlıurfa, Turkey

**Keywords:** AI based diagnosis, Deep learning, Object detection, Orthodontically induced root resorption, Panoramic radiography, Pose estimation, Root resorption

## Abstract

**Objectives:**

This study aimed to develop and compare two YOLOv12-based deep learning models—object detection and pose estimation—for the automatic classification of orthodontically induced external apical root resorption (OIEARR) using panoramic radiographs.

**Materials and methods:**

A total of 624 panoramic radiographs obtained from 312 patients aged 10–18 who underwent at least 12 months of fixed orthodontic treatment were retrospectively analyzed. Each maxillary central and lateral incisor was graded for OIEARR severity on a 4-point scale (Grade 0 to Grade 3) by two experienced orthodontists serving as the ground truth. Two YOLOv12-based models were trained: an object detection (OD) model for regional analysis and a pose estimation (PE) model for anatomical landmark localization. Both models were trained and validated on annotated panoramic images and evaluated using accuracy, precision, recall, specificity, F1-score, confusion matrix, and ROC-AUC.

**Results:**

The PE model outperformed the OD model across all evaluation metrics, demonstrating superior performance in detecting OIEARR. Specifically, the PE model achieved a weighted F1-score of 0.88, compared to 0.60 for the OD model. It also showed higher accuracy (0.93 vs. 0.78), precision (0.88 vs. 0.64), and recall (0.88 vs. 0.59), confirming its robustness in root resorption classification. Particularly in Grade 1 and Grade 2 resorption categories, the PE model demonstrated markedly superior classification performance (F1 = 0.85 and 0.88, respectively), while maintaining excellent detection in Grade 3 cases (F1 = 0.95). Confusion matrix analysis revealed that most misclassifications occurred between neighboring grades. ROC-AUC values for the PE model were consistently high (0.90–0.99), indicating strong discriminative ability across all resorption stages.

**Conclusions:**

The YOLOv12x PE model offers a reliable and sensitive tool for detecting varying degrees of root resorption on panoramic radiographs. Its fine-grained anatomical localization capabilities provide an advantage for early diagnosis, making it a promising approach for clinical decision support in orthodontics.

## Introduction

Orthodontically induced external apical root resorption (OIEARR) is one of the major complications of orthodontic treatment due to progressive potential and irreversible nature. It is considered to occur when the forces at the apex during treatment exceed the resistance and reparative ability of the periapical tissues [1–3]. This is an undesirable consequence of orthodontic treatments [4] and is often a source of concern for clinicians [5, 6]. Despite the absence of consensus regarding the aetiology of OIEARR, a multifactorial and complex problem, a number of factors have been reported as potentially contributory to the development of the condition, including individual predisposition, genetic susceptibility, anatomical features and orthodontic mechanotherapy [7, 8].

More than 90% of orthodontically treated teeth have been reported to have OIEARR in histological studies [9, 10]. Using diagnostic radiographic techniques, mild to moderate OIEARR has been reported in 40%–60% of cases [11]. However, if not diagnosed in time and its progression is not prevented, severe cases of root resorption are encountered and have been reported in the range of 1%–5% [8]. In most cases of mild OIEARR, tooth survival and normal function are unaffected [8, 12, 13], but in severe cases, root shortening of more than 4 mm or one third of the root [8]can threaten tooth survival [12, 14, 15]. Therefore, although the mild form is clinically less significant, it is imperative to diagnose the condition before it progresses to a more severe stage of OIEARR.

Although OIEARR can occur in any tooth in the mouth, it is most commonly observed in the upper incisors [16, 17]. The asymptomatic character of the condition requires vigilance on the part of the clinician. As mild to moderate OIEARR is not accompanied by symptoms, it is typically diagnosed through the analysis of panoramic or periapical radiographs, which are commonly obtained during orthodontic treatment. Panoramic radiographs, which provide a comprehensive view of the entire maxillomandibular complex, including the temporomandibular joints, are extensively utilised as an orthodontic diagnostic instrument for the identification of OIEARR [18, 19]. Although panoramic radiography is not considered the optimal imaging technique for diagnosing and monitoring OIEARR due to distortion in the incisor area, a recent study found that 97.5% of orthodontists prefer it as the most common pre-treatment screening method for OIEARR [19].

In recent years, there has been an increase in computer-aided diagnostic procedures based on artificial intelligence (AI), particularly in dental applications that require radiographic evaluation [20–22]. As a one of these procedures, the YOLO family, is widely favored for real-time object detection due to its ability to process entire images in a single pass, offering both high speed and accuracy [23]. This efficiency is especially valuable in medical imaging, where timely and reliable diagnostics are critical [24]. In recent years, the YOLO architecture has been increasingly utilized in dental radiographic analysis for a variety of diagnostic tasks, including caries detection, tooth localization, mandibular fracture identification, the assessment of periodontal bone loss and cephalometric landmark detection [25–29]. Over the years, YOLO algorithms have become more effective at processing input images and improving feature integration across different scales. Finally, architectural advancements were introduced in YOLOv12 in 2025 to improve training stability and model convergence [30]. The object detection model is trained to find and classify objects in an image. This process typically involves training a model to detect an object by learning to classify regions in an image, and then drawing a bounding box around the detected object [31]. Conversely, the primary objective of pose estimation is to identify objects in each frame and categorise the different poses in an image [32]. It is unclear how combining these two complementary modelling approaches within the same diagnostic workflow will aid the detection of OIEARR, particularly given the importance of accurately determining the affected area and its structural features.

In a few studies in the literature on OIEARR, artificial intelligence supported systems have been used to detect root resorption on cone-beam computed tomography (CBCT). These studies reported that deep learning-based methods using CBCT images provide reliable and automatic tools for detecting OIEAR [20–22]. Unlike panoramic radiographs, CBCT is not usually used for diagnosing OIEARR but can serve as a valuable investigative tool in severe cases. Conversely, panoramic radiographs, despite certain disadvantages, are frequently used by clinicians during orthodontic treatment, as mentioned previously. Therefore, the ability to detect OIEARR on panoramic radiographs at an early stage is essential, before reaching advanced stages. However, in clinical practice, the mild resorption of root tissue can be challenging to discern on panoramic radiographs. The potential application of AI in this domain is a subject of considerable interest. A thorough review of the existing literature does not reveal any studies that demonstrate the effectiveness of AI in the detection of OIEARR in panoramic radiographs.

The objective of the present study is to evaluate the efficiency of deep learning-based AI techniques in the automated diagnosis of OIEARR of upper incisors in panoramic radiographs. The contribution of reliable results to human assessment will provide a significant aid in the identification of cases of OIEARR that have been overlooked.

## Materials and methods

### Ethical approval and patient selection

**Fig. 1:**
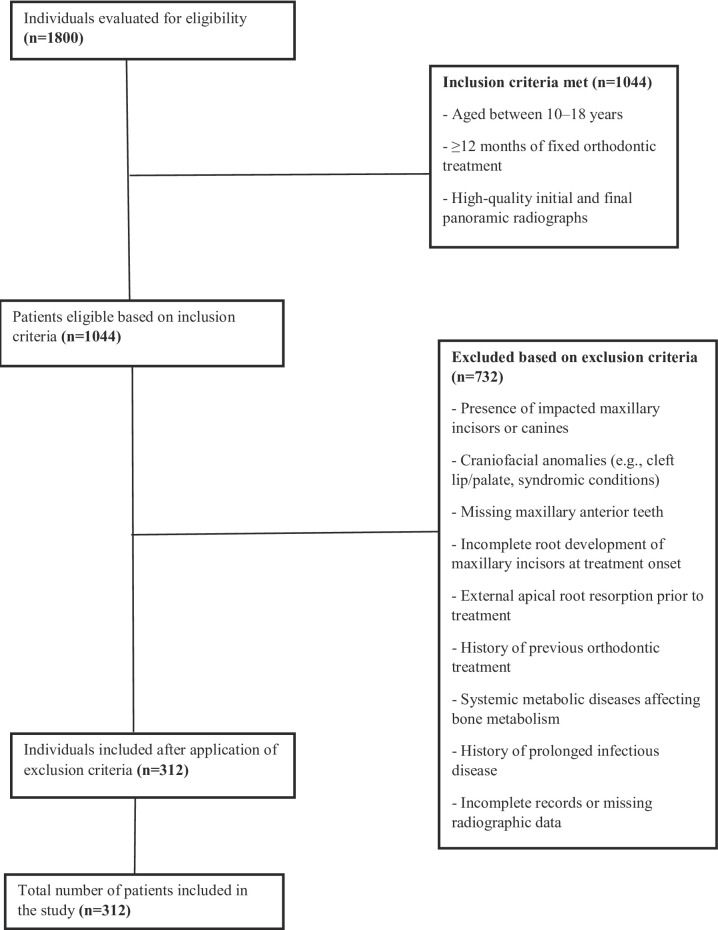
Flowchart of patient selection based on inclusion and exclusion criteria

This retrospective study received ethical approval fromİnönü University Non-Interventional Clinical Research Ethics Committee (Approval Number:2025/7540, Date: 04/30/2025), confirming adherence to ethical guidelines. The study retrospectively analyzed panoramic radiographs of patients aged 10 to 18 years who had undergone at least 12 months of fixed orthodontic treatment at the Department of Orthodontics, Faculty of Dentistry, İnönü University, between 2010 and 2025. A detailed overview of the inclusion and exclusion process applied to the selection of patients based on panoramic radiographs is presented in Fig. 1.

### Power analysis

A priori power analysis was performed to ensure that the dataset was statistically sufficient. Vemu et al. [33] demonstrated that the model’s performance significantly exceeded the random guess level (~ 33%) with a binomial test at *p* < 0.001. Krois et al. [34] compared the accuracy of a CNN model with that of experienced dentists in their study to detect periodontal bone loss on panoramic radiographs. The researchers used G*Power to calculate the sample size to determine a 3% difference, assuming a bone loss detection accuracy of ~ 80% for dentists and ~ 83% for the model. In our study, a power analysis was performed using G*Power 3.1.9.7 software to assess whether our model produced a 5% performance difference compared to random guessing. Because the random classification accuracy for a four-class problem was 25%, the null hypothesis was defined as H₀ = 0.25. Analysis using the exact binomial test (one-tailed) with parameters g = 0.05, α = 0.05, and power = 0.80 indicated that at least 494 samples were needed for statistically significance. When the both training and testing data were added, this number increases to 618, and accordingly, 624 panoramic radiographs were included in the present study. Furthermore, because our YOLOv12-based model is supported by transfer learning, dense boosting, and high-quality annotations, this dataset is technically sufficient for deep learning training.

### Observer-based clinical grading of external apical root resorption

Before the development and evaluation of the deep learning models, a clinical reference standard was established through independent radiographic assessments. Two experienced orthodontists (S.Ö and M.T), each with over five years of clinical expertise, collaboratively evaluated the panoramic radiographs obtained at the initiation and completion of orthodontic treatment.

External apical root resorption (EARR) was graded in accordance with the criteria described by Sharpe et al. [35], whereby: (0) no apical root resorption was present; (1) slight blunting of the root apex was observed; (2) moderate resorption extended beyond blunting and up to one-third of the root length; and (3) severe resorption exceeded one-third of the root length.

All radiographs were assessed in a joint session, and the final resorption grade for each maxillary central and lateral incisor was determined through consensus. This consensus-based grading system constituted the clinical ground truth against which the diagnostic performance of the deep learning models was compared.

### Data Preparation and annotation for deep learning

Digital panoramic radiography records of all patients were obtained using the same device (Planmeca proline XC, 00880, Helsinki, Finland) with exposure parameters of 66 kV, 5.0 mA, and 18 s. The dataset preparation involved two different annotation strategies to identify key anatomical landmarks of the maxillary incisors, including the incisal edge, cervical margin, and root apex. Anatomical landmarks corresponding to these regions were manually annotated using the Computer Vision Annotation Tool (CVAT), following a standardized labeling protocol to ensure consistency across the dataset. In this context, the following two YOLOv12 deep learning models were utilised in the present study.

**Fig. 2:**
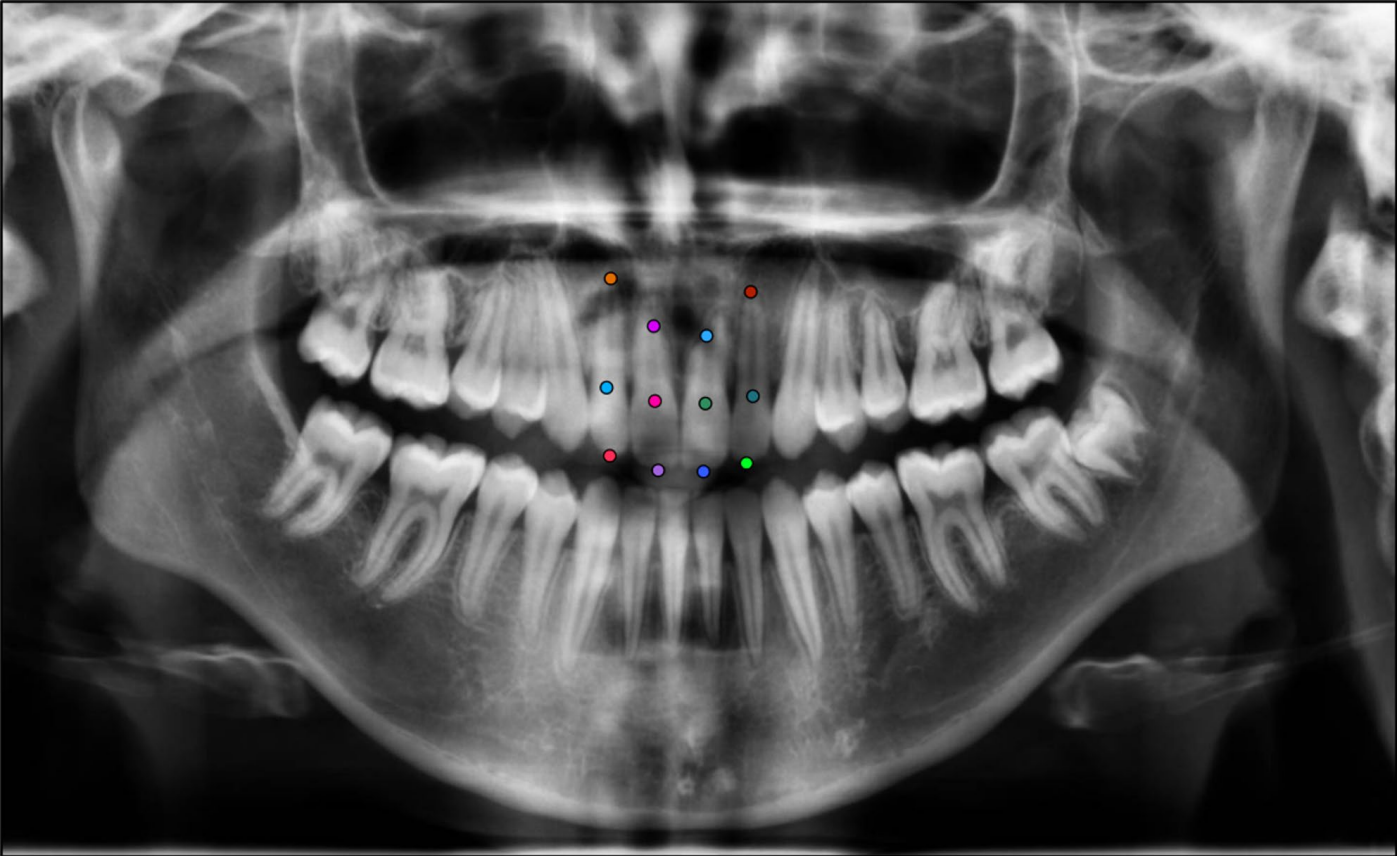
Illustration of the 12 key points used in the YOLOv12x pose estimation model for identifying root resorption in maxillary incisors

*1- Pose Estimation (PE) Model (YOLOv12x-pose):* PE model was utilized to identify three anatomical landmarks—the incisal edge, cervical margin, and root apex—on the maxillary central and lateral incisors using panoramic radiographs. A total of ‘12 key points’ per patient were labeled (Fig. 2). For each incisor, root length was calculated as the distance between the predicted cervical and apical points, enabling the quantification of external apical root resorption. The percentage reduction in root length between pre-treatment and post-treatment radiographs was automatically computed, providing objective measurements for comparison with manual clinical assessments.

**Fig. 3:**
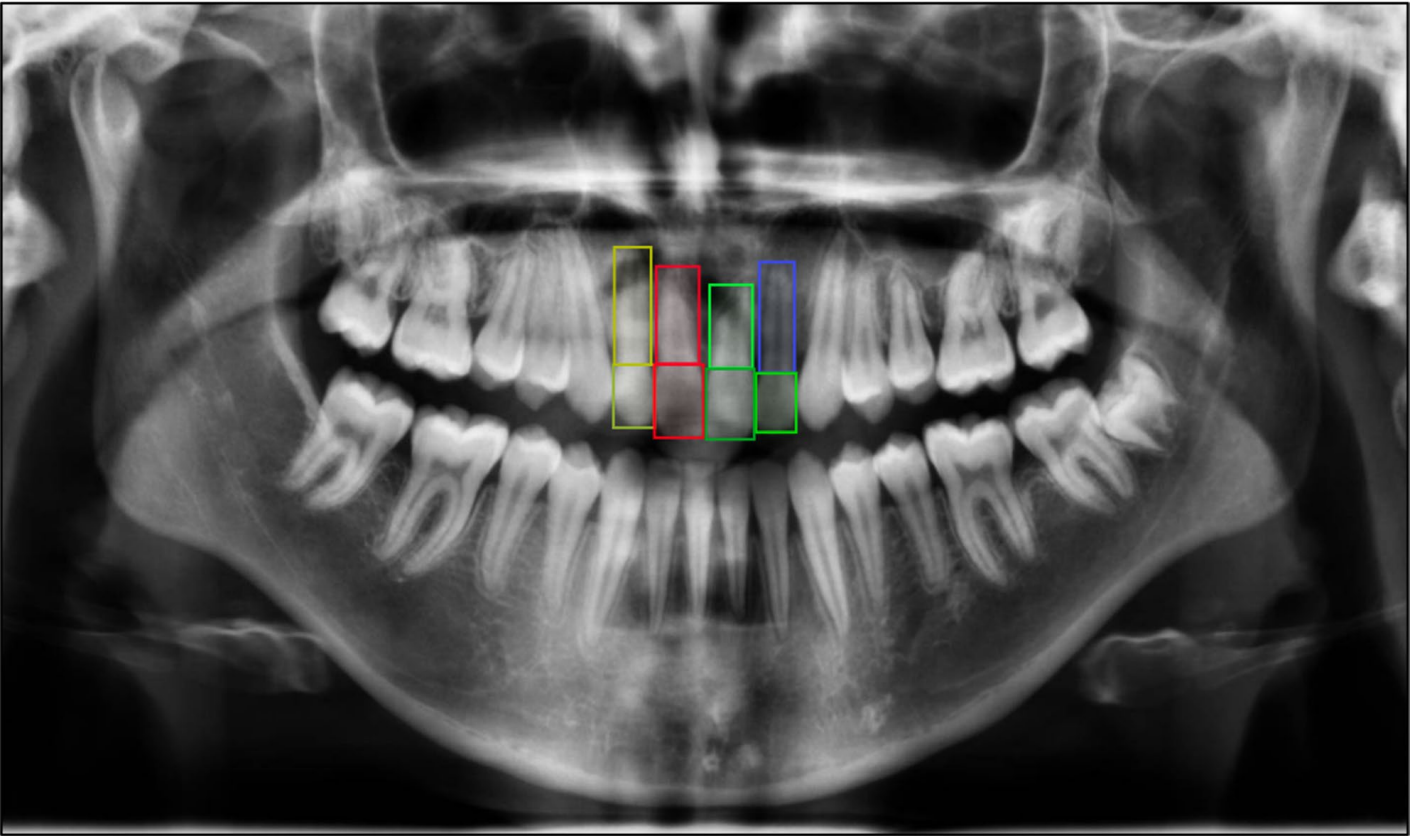
Illustration of the eight bounding boxes used in the YOLOv12x object-detection model for identifying root resorption in maxillary incisors

*2- Object Detection (OD) Model (YOLOv12x):* OD model was applied to localize the crown and root regions of the maxillary central and lateral incisors on panoramic radiographs. A total of 8 bounding boxes per patient were labeled (Fig. 3). Rectangular regions of interest (ROIs) encompassing the incisal edge, cervical margin, and root apex were generated for each tooth at both the baseline and post-treatment stages. Root resorption was quantified by calculating the percentage reduction in root length between the two time points. Automated measurements were recorded for each tooth to enable comparison with manual clinical assessments.

### Training of the deep learning models

**Fig. 4:**
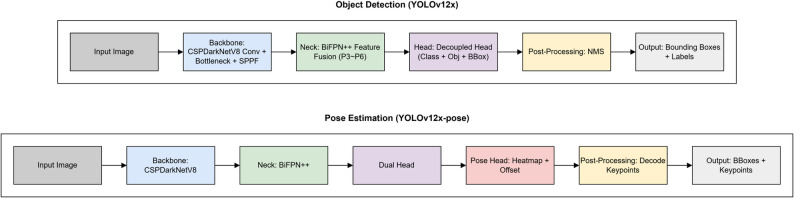
Architectural workflow of the YOLOv12x-based object detection (top) and pose estimation (bottom) models used in this study

Two separate deep learning models were developed based on the Ultralytics YOLOv12 architecture. Both the OD and PE models were built upon a shared backbone (CSPDarkNetV8) and feature fusion neck (BiFPN++), enabling multi-scale feature extraction. In the OD pipeline, a decoupled head structure was employed for simultaneous class prediction and bounding box regression, followed by non-maximum suppression (NMS) to finalize the output regions. In the PE pipeline, a dual head structure was used to generate heatmaps and offset vectors for predicting the coordinates of anatomical landmarks. While the OD model provided coarse regional localization, the key point-based PE model was designed to yield fine-grained coordinate-level outputs for anatomically relevant points. The PE model was therefore hypothesized to offer greater precision in detecting root resorption patterns, particularly in cases involving small or complex morphological changes. The architectural structure of the YOLOv12-based OD and PE models implemented in this study is summarized in Fig. 4.

This study analyzed a total of 624 panoramic radiographs obtained from 312 patients who had undergone fixed orthodontic treatment. The dataset was divided into training (*n* = 496, 80%), validation (*n* = 64, 10%), and test (*n* = 64, 10%) sets. To generalize the model, all training and validation datasets were used during model training, regardless of whether they were taken before or after treatment. However, in the test dataset, the model developed for external root resorption classification was applied separately to pre- and post-treatment radiographs, and the results were converted into grades using the developed technique. Therefore, the total number of test radiographs (64) consists of 32 pre-treatment and 32 post-treatment radiographs. Thus, the test dataset includes radiographs from 32 patients, and since 4 teeth were examined in each radiograph, a total of 128 teeth were examined.

Photometric augmentations were used to ensure that the model could make accurate predictions under different brightness conditions, color variations and contrast levels; geometric augmentations were used to ensure that the model could recognize objects in different directions, positions and sizes; and distortion & noise augmentations were used to prevent the model from losing performance in low-quality, blurry or noisy data. Prior to input, all image pixel values were normalized from a range of 0–255 to 0–1.

Both models were implemented in the PyTorch framework and trained on a high-performance workstation equipped with an Intel Core i9-12900 K processor, 128 GB DDR5 RAM, and an NVIDIA Quadro RTX A6000 GPU. Both models were trained using the same hyperparameters. An early stopping mechanism was used to prevent over-learning. The model that gave the best result on the validation set was saved and used on the test set. The hyperparameters used and their values ​​are given in the table below (Table 1).

**Table 1:** Hyperparameters used in training the deep learning models and their corresponding values

Hyperparameters	Value
Training Configuration	
Epochs	1000
Early stopping patience	30
Optimizer	AdamW
Momentum	0.9
Batch size	4
Target image size	640
Weight decay	5 × 10^− 4^
Warm-up epochs	3
Warm-up momentum	0.0
Warm-up bias learning rate	0.0
Initial learning rate	10^− 3^
Final learning rate	10^− 5^
Learning rate schedule	Cosine decay
Loss parameters	
Box loss gain	7.5
Classification loss gain	0.5
Distribution focal loss loss gain	1.5
Pose loss gain	12.0
Keypoint objectness loss gain	2.0
Augmentation parameters	
HSV saturation	0.7
HSV value	0.4
HSV hue	0.015
Degrees	1.0
Translate	0.1
Scale	0.5
Blur	Limit=(3, 7)
MedianBlur	Limit=(3, 7)
ToGray	Channels = 3, Method=weighted_average
CLAHE	Limit=(1, 4), Grid Size=(8, 8)

### Deep learning-based measurement of external apical root resorption

Following model inference, root length predictions were generated separately by the PE and OD models. For the PE model, root length was estimated based on the linear distance between the predicted incisal edge and root apex coordinates. For the OD model, the vertical dimension of each bounding box, encompassing the crown and root regions, was used to estimate root length.

To reduce the impact of magnification, distortion, and projection-related variations encountered in panoramic radiographs on measurement results, a ratio-based and normalized calculation approach has been integrated into the measurement process. In this context, coordinates from YOLOv12-based models have been normalized according to radiograph dimensions. Furthermore, root resorption calculations are based not on absolute pixel or millimeter lengths, but on time-dependent proportional changes within the same tooth. Normalization was performed as follows:

$$\:Normalized\:X\:coordinate=\frac{X\:coordinate}{Radiograph\:width}$$

$$\:Normalized\:Y\:coordinate=\frac{Y\:coordinate}{Radiograph\:height}$$

To compensate for possible scale and projection differences between pre- and post-treatment images, root length measurements have been normalized based on crown dimensions. Assuming that root resorption does not affect crown length, and therefore crown length remains the same in pre- and post-treatment radiographs, the post-treatment root length was corrected as follows:

$$\:Correction\:Factor=\frac{Initial\:crown\:length}{Post\:Crown\:lenght}$$

$$\begin{aligned} & Corrected\:final\:root\:lenght = \frac{{Expected\:final\:root\:lenght}}{{Correction\:factor}} \\ \end{aligned} $$

Following this correction, root resorption was calculated using the pre-treatment root length of the same tooth as a reference:

$$\begin{aligned} & Root\:resorption\:\left( \% \right)= \frac{{Initial\:root\:lenght - corrected\:final\:root\:lenght}}{{Initial\:root\:lenght}}\quad \times 100 \\ \end{aligned} $$

Due to potential variations in root angulation, patient positioning, and focal trough alignment, measurement error was calculated in both OD and PE models using teeth from the same patients that were classified as grade 0 root resorption. Accordingly, in the measurement error analysis, teeth that were clinically graded as Grade 0 (no detectable resorption) by the orthodontic experts were assumed to have a true resorption value of 0%, and these cases were used as a reference set to quantify the intrinsic measurement error of the developed models. The predicted resorption percentages generated by both the OD–based bounding box model and the PE–based keypoint detection model were directly compared against this reference.

Specially developed Python scripts applied this formula autonomously to determine the percentage reduction in root length between the pre-treatment and post-treatment stages. Subsequently, the predicted continuous resorption percentages were categorized into discrete grades based on the classification system proposed by Sharpe et al. [35], allowing direct comparison with the clinical consensus gradings.

### Performance metrics for evaluating the deep learning models

To evaluate the classification performance of the deep learning models on the test set, confusion matrices were constructed. These matrices enabled a detailed comparison between the model outputs and the corresponding ground-truth labels, providing insight into classification accuracy. Model performance was assessed using key evaluation metrics, including Accuracy, Precision, Recall, and F1 Score, which were calculated based on the numbers of True Positives (TP), False Negatives (FN), False Positives (FP), and True Negatives (TN).

Resorption percentage values generated by YOLOv12-based OD and PE models were converted into 0–3 grade categories using clinically defined thresholds. Subsequently, ROC analysis was performed using the one-vs-rest method, where each grade was considered the positive class and the remaining grades were considered the negative class. True positive, false positive, false negative, and true negative values were calculated based on ground truth orthodontist evaluations. The ROC curve was plotted by examining the relationship between sensitivity (recall) and false positive rate, and the area under the curve (AUC) was calculated. Values closer to 1.0 indicated stronger discrimination performance.

**Fig. 5:**
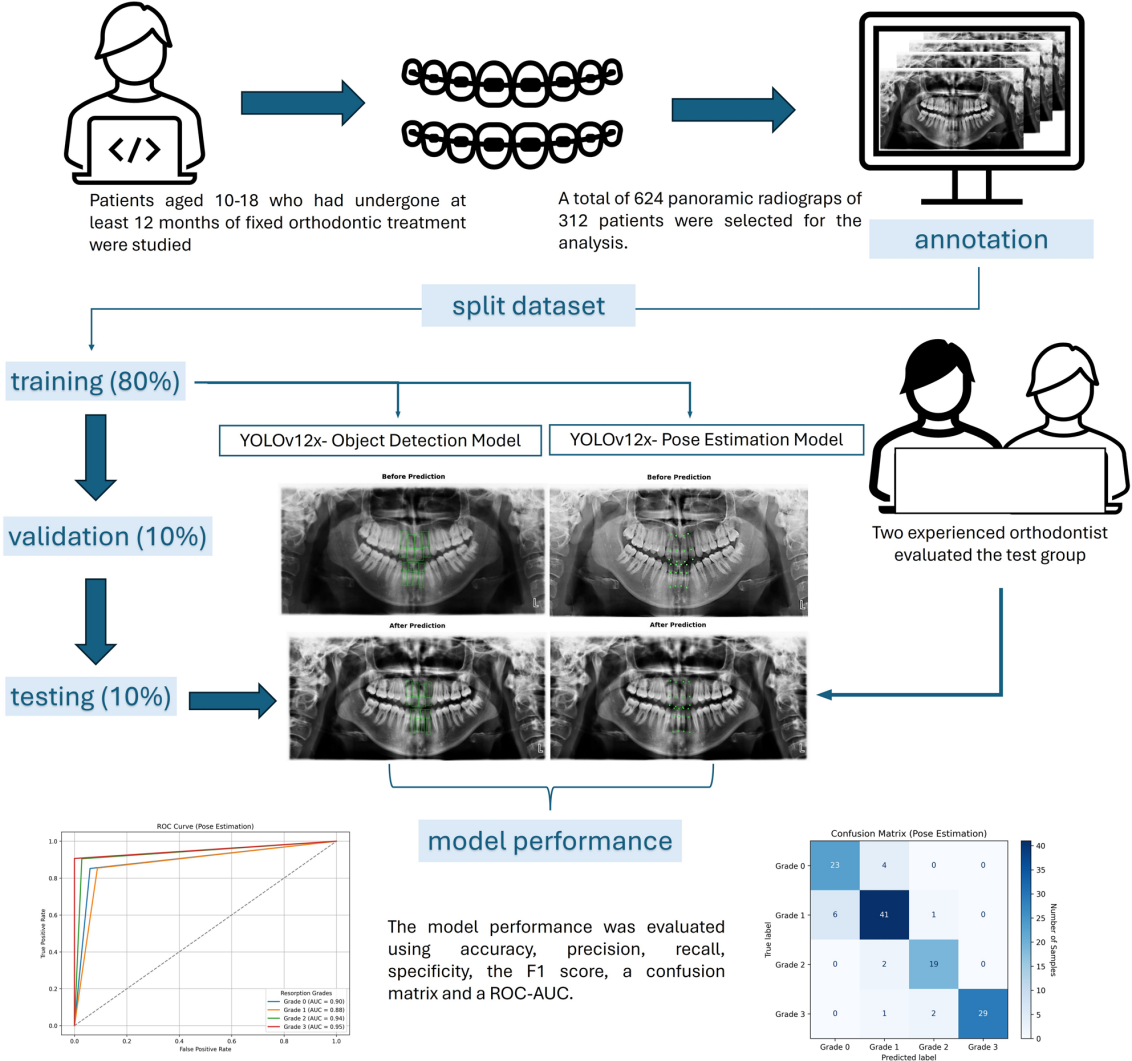
Flowchart summarizing the study workflow and methodological steps

A flowchart summarizing the overall workflow and methodological steps of this study is presented in the graphical abstract (Fig. 5).

### Measurement error

To assess the reproducibility of the clinical grading, a randomly selected subset comprising 20% of the panoramic radiographs was re-evaluated by the same two orthodontists one month after the initial assessment. The re-evaluation was performed through consensus, following the same standardized clinical criteria. The consistency between the initial and repeated consensus-based gradings was assessed using the intraclass correlation coefficient (ICC).

Following the assessment of clinical grading reproducibility, the methodological measurement error associated with the deep learning–based root length estimation was evaluated using teeth classified as Grade 0.

## Results

An intraclass correlation coefficient (ICC) of 0.989 indicated excellent reproducibility between baseline and one-month follow-up measurements, based on consensus evaluations performed by two experienced orthodontists. According to the results of the methodological measurement error analysis, the mean error and standard deviation were 3.13% and 5.85% for the OD model, and 0.74% and 1.81% for the PE model, respectively.

The classification performance of both models was evaluated using accuracy, precision, recall, specificity, and F1 score across all resorption grades (Table 2). The PE model consistently outperformed the OD model in both overall metrics and grade-specific classifications.

**Table 2:** Comparison of classification performance metrics for YOLOv12-based pose estimation and object detection models across root resorption grades

	Bounding box (YOLOv12-pose estimation model)					Keypoint (YOLOv12-object detection model)				
	Grade 0	Grade 1	Grade 2	Grade 3	Weighted average	Grade 0	Grade 1	Grade 2	Grade 3	Weighted average
Accuracy	0.75	0.72	0.81	0.90	0.78	0.92	0.98	0.85	0.91	0.93
Precision	0.44	0.66	0.42	0.91	0.64	0.79	0.85	0.86	0.99	0.88
Recall	0.67	0.52	0.52	0.66	0.59	0.85	0.85	0.90	0.91	0.88
Specificity	0.77	0.84	0.86	0.98	0.86	0.94	0.91	0.97	0.99	0.95
F1 score	0.53	0.58	0.47	0.76	0.60	0.82	0.85	0.88	0.95	0.88

For Grade 0 (no apical root resorption), the PE model demonstrated strong classification performance, achieving 0.92 accuracy, 0.79 precision, 0.85 recall, and an F1 score of 0.82. In contrast, the OD model yielded lower values, with 0.75 accuracy, 0.44 precision, 0.67 recall, and an F1 score of 0.53.

For Grade 1 (slight blunting of the root apex), the PE model again exhibited superior performance with 0.98 accuracy, 0.85 precision, 0.85 recall, and an F1 score of 0.85. The OD model, however, showed reduced sensitivity, with 0.72 accuracy and an F1 score of 0.58,

For Grade 2 (moderate resorption extending beyond blunting and up to one-third of the root length), the PE model maintained robust performance, achieving 0.85 accuracy, 0.86 precision, 0.90 recall, and an F1 score of 0.88. In comparison, the OD model struggled significantly in this class, yielding only 0.81 accuracy, 0.42 precision, 0.52 recall, and an F1 score of 0.47.

For Grade 3 (severe resorption extending beyond one-third of the root length), both models achieved relatively high accuracy. The PE model achieved 0.91 accuracy, 0.99 precision, 0.91 recall, and an F1 score of 0.95, showing a nearly perfect classification capability. The OD model also performed reasonably well with 0.90 accuracy, 0.91 precision, 0.66 recall, and an F1 score of 0.76.

For the overall classification, the weighted average performance of the PE model reached 0.93 accuracy, 0.88 precision, 0.88 recall, 0.95 specificity, and an F1 score of 0.88. In contrast, the OD model yielded a weighted accuracy of 0.78, precision of 0.64, recall of 0.59, specificity of 0.86, and an F1 score of 0.60.

**Fig. 6:**
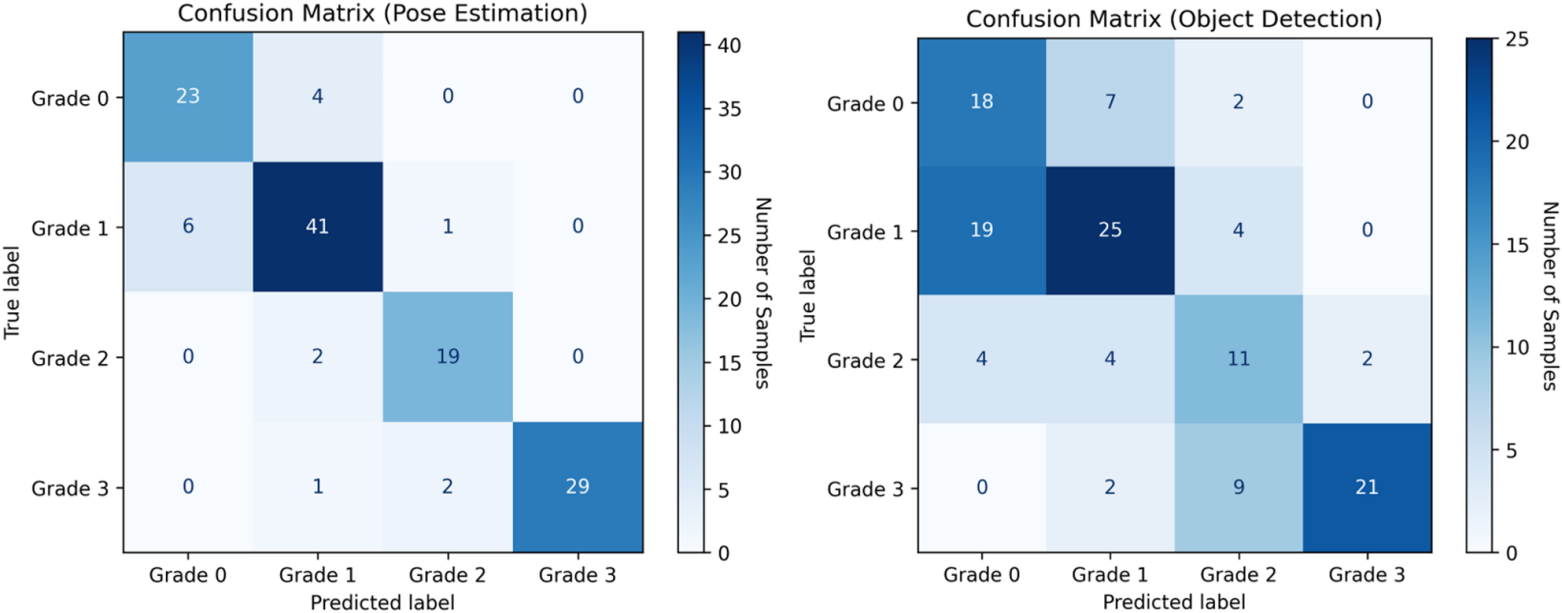
Combined confusion matrices for the four root resorption grades evaluated using YOLOv12-based object detection and pose estimation models. Diagonal values represent correctly classified instances, while off-diagonal values indicate misclassifications between different resorption grades

Figure 6 presents representative confusion matrices for both the PE and OD models, illustrating the AI models’ performance across resorption grades. Diagonal elements indicate correctly classified instances (TP and TN), with higher concentrations reflecting stronger classification accuracy. Off-diagonal values represent misclassifications (FP and FN), often occurring between adjacent grades. In the OD model, misclassification was most prominent between Grade 0 and Grade 1, suggesting difficulties in detecting early-stage resorption. Grade 2 also exhibited confusion with both Grade 1 and Grade 3. In contrast, the PE model demonstrated a more diagonally dominant matrix with minimal misclassification, particularly achieving high accuracy in Grade 3 classification and showing limited overlap in other categories.

**Fig. 7:**
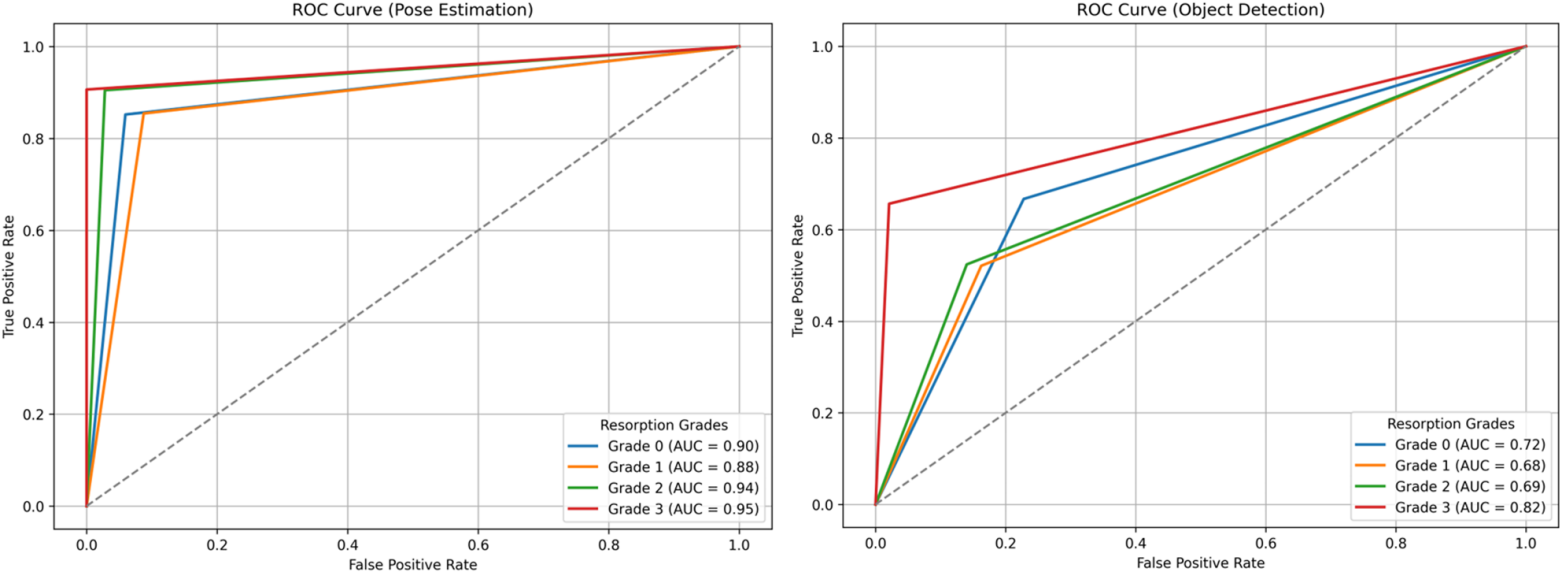
Receiver Operating Characteristic (ROC) curves for the YOLOv12-based object detection and pose estimation models. The curves illustrate the models’ diagnostic performance across four root resorption grades. AUC values closer to 1.0 indicate higher classification reliability and better discrimination capability

Figure 7 displays the ROC curves for both the PE and OD models, illustrating their discriminative performance across resorption grades.AUC, representing the overall ability of the model to distinguish between classes, approaches 1.0 for high sensitivity and specificity, while values near 0.5 indicate poor discrimination. In the PE model, Grade 3 (AUC = 0.98) and Grade 0 (AUC = 0.96) exhibited near-perfect classification, while Grade 1 and Grade 2 also achieved strong diagnostic performance (AUC = 0.94 and 0.93, respectively). In contrast, the OD model demonstrated lower AUC values across all classes, particularly in Grade 1 (AUC = 0.76) and Grade 2 (AUC = 0.79), indicating a reduced ability to distinguish between adjacent resorption levels. These results confirm the superior discriminative power of the PE-based approach in differentiating between varying severities of root resorption.

## Discussion

OIEARR is a recognised adverse effect of orthodontic treatment, but it is hard to identify [8]. It is often found on routine panoramic radiographs, so more accurate tools are needed. This study aimed to automatically detect and classify OIEARR on panoramic radiographs using an AI based approach. The number of studies conducted on OIEARR in panoramic radiographs in the literature is quite limited [36] and our study provides a detailed methodology and comparative analysis as an article.

In the literature, it has been reported that orthodontists predominantly rely on radiographic imaging techniques to evaluate OIEARR [37]. While periapical and panoramic radiographs are widely used, both have limitations. Periapical images are prone to inter-operator variability due to standardization difficulties, while panoramic radiographs may suffer from magnification errors and image distortion. Some studies suggest that panoramic radiographs overestimate root loss by approximately 20% compared to periapical radiographs [38], but it is still routinely used for screening before and during orthodontic treatment [39]. Currell et al. [19] reported that panoramic radiograph remains the most commonly used imaging modality for the detecting OIEARR contrasting with reports that periapical radiographs are more frequently preferred for the screening of OIEARR [38, 39].

While CBCT provides higher diagnostic accuracy, its routine use is limited due to higher radiation dose and cost. Low-dose protocols reduce exposure, but CBCT still involves greater radiation compared to 2D imaging [40, 41]. Previous studies also stated that it would be a good clinical practice to take a panoramic radiograph 6–12 months after the start of fixed treatment because of its significant association with the OIEARR grade at the end of treatment [18, 38]. Additionally the early detection of pre-existing resorption is of significant clinical importance for treatment planning and deciding long-term outcomes. As CBCT is not routinely acquired in clinical practice, the ability to detect pre-existing resorption on panoramic radiographs is substantially helpful to practitioners. The proposed methodology offers a valuable screening instrument for initial and quantitative assessment of root resorption in contexts where CBCT is not readily accessible or financially viable and involves significantly higher equipment. The use of panoramic radiographs in this study was a practical, accessible and well-founded decision that aligns with ethical principles, reflects real-world clinical practice and provides a feasible foundation for the application of AI-driven diagnostic tools.

Our study used the same imaging device and operator for all panoramic radiographs to minimize inter-device and inter-operator variability. This methodological consistency aimed to control one of the major sources of measurement error: variations in head positioning. Previous studies have shown that even minor deviations in head position can significantly affect anterior tooth length measurements on conventional panoramic images [42]. While 3D panoramic radiography has been proposed as a method to mitigate such errors, we attempted to address this limitation by standardizing both the imaging protocol and the operator technique. This controlled approach strengthens the internal validity of our findings and supports the reliability of the AI-based root length assessments derived from conventional panoramic radiographs.

The upper incisors are the teeth most affected by OIEARR [8, 11, 16] and are of high aesthetic and phonation importance. In contrast to multi-rooted posterior teeth, which exhibit significant morphological variability, upper incisors with relatively straight roots and fully developed roots are more suitable for evaluation. Therefore, in line with previous studies [2, 5, 12–15] the current study focused on assessing only the upper incisors to avoid the morphological complexity of posterior teeth from influencing the results.

There is no consensus in the literature on the best way to measure OIEARR, whether in millimetres or as a percentage of root loss. According to Levander and Malmgren [43], root resorption is classified as mild when less than 2 mm or one-third of the root length is affected; moderate when it’s over 2 mm but below one-third; and severe when it’s over one-third. In contrast, some researchers have defined severe resorption as a loss greater than 5 mm [44]. Additionally, several studies suggest that using a percentage-based evaluation rather than absolute millimetric values may be more clinically meaningful when assessing the severity of OIEARR [15]. Based on this rationale, we used a percentage-based grading system to measure OIEARR.

This study utilized the advanced YOLOv12 framework. YOLOv12 introduces a new architecture centered around attention mechanisms, moving away from the traditional CNN-based designs of earlier YOLO models. Despite this shift, it preserves the real-time inference speed critical for many use cases, while achieving state-of-the-art accuracy through innovative advancements in both attention strategies and overall network design. In both models, the YOLOv12 structure was preferred due to its improved capacity and detailed feature extraction for tasks requiring high accuracy in complex scenes.

Karamüftüoğlu’s findings are consistent with our analysis. The RT-DETR-X model achieved the highest overall accuracy (0.434), but the YOLOv12x model had a superior balance. YOLOv12x’s precision of 0.442 almost matches the 0.440 of RT-DETR-X and significantly surpasses the 0.326 of RT-DETR-L. This higher precision means fewer false positives. However, YOLOv12x’s lower sensitivity of 0.333 means more challenging lesions may be missed. Its lightweight architecture and accuracy make it ideal for high-speed applications [45]. In contrast Saber et al. applied YOLOv8, YOLOv11 and YOLOv12 one stage OD for automated apical periodontitis detection on periapical radiographs, showing the best results for YOLOv12m (89.1% precision), but also the strong performance of YOLOv11m (F1 score: 87.1%, better at detecting early-stage lesions [PAI scores 1 and 2]). These results confirm that lightweight YOLO models can identify specific pathologies, so are well suited for clinical screening and diagnosis [30]. In this context, our study is the first to examine OIEARR using the YOLOv12x and YOLOv12x-pose model in panoramic radiographs.

While OD and PE methods are deep learning based and effective in image segmentation and classification, their structural differences directly impact model performance, especially in relation to the importance of morphological details.

For the OD model, which estimates root length based on the vertical dimension of the predicted bounding boxes encompassing both crown and root regions, the mean absolute error was 3.13%, with a standard deviation of 5.85%. The model’s higher error and variability can be attributed to several factors. Bounding box predictions include non-anatomical background regions and are sensitive to minor inaccuracies in box localization, particularly at the apical and incisal boundaries. Second, they’re susceptible to distortion, tooth inclination and variations in crown–root overlap. Consequently, minor deviations in box height may translate into amplified errors in the derived root length ratios. The PE model, predicting anatomical landmarks to compute root length, demonstrated higher measurement stability, with a mean error of 0.74% and standard deviation of 1.81%, indicating both higher accuracy and lower dispersion across samples. The PE model’s superior performance is due to its anatomically constrained formulation, which directly localises keypoints, making the measurement process less sensitive to noise and effects. Normalised coordinates and crown-based correction stabilise distances, especially when comparing pre- and post-treatment images. From a clinical perspective, these findings indicate that the PE-based approach is more reliable in measuring minimal or early-stage root resorption, where small changes must be detected with precision. However, bounding box–derived root length measurements may be less optimal for detecting subtle changes.

When the confusion matrices are examined, a significant performance difference is observed between the two different approaches. In the OD model, a significant portion of the cases labeled Grade 1 were misclassified as Grade 0. This situation shows that the early stages of resorption were mixed with low levels and the model could not distinguish the class boundaries sufficiently and indicating substantial confusion with Grade 0 cases and limited ability to detect early-stage resorption. In addition, similar shifts to Grade 1 and Grade 3 were observed in the Grade 2. These error distributions can be associated with the fact that the OD model bases its classification decisions only on the general image information within the bounding boxes and cannot adequately represent the local morphological details. On the other hand, the PE model was able to make a clearer and more error-free distinction in each class. Only 6 examples were classified as Grade 0 in the Grade 1 class, and the cross-mixing in the other classes was extremely low. This suggests that the PE model was more effective in avoiding false positives while preserving sensitivity in identifying healthy roots. For Grade 2 the results underscore the PE model’s enhanced capacity to capture moderate structural changes in the root apex region. For the Grade 3, the model showed a hit rate close to 100%. Despite similar accuracy, the PE model’s higher recall and F1 score highlight its reduced false negative rate in detecting severe cases. These findings clearly demonstrate that the PE model offers a more balanced and clinically reliable classification approach across all stages of root resorption, with particularly superior performance in early and moderate grades where diagnostic sensitivity is most critical.

Chen et al. [46] used an R-CNN architecture with a OD approach, achieving high accuracy in tooth localization; however, tooth numbering required additional postprocessing to improve classification performance. Tichy et al. [47]. and Pérez et al. [48] reported low expert consensus and frequent misclassification in low-contrast carious lesions using OD annotations, highlighting the limitations of OD based models in capturing subtle morphological details. Similarly, in the present study, the OD model showed limited ability to distinguish early-stage root resorption (Grades 0–1). Conversely, the PE model had high accuracy, AUC, and F1 scores, especially in critical classes. This shows the benefit of architectures that effectively capture localised morphological features in improving reliability.

In the literature, deep learning approaches based on localized information for the automatic classification of root resorption remain limited, positioning the present study as a pioneering contribution in this field [20–22, 49–51]. The accurate classification of early-stage resorptions, particularly Grade 0 and 1, is of great importance for clinical management and patient monitoring. In this context, the limited sensitivity of OD models to morphological details highlights the potential need for future systems to evolve into hybrid models supported by additional layers such as segmentation [52]. This study demonstrates that architectures with higher capacity for detailed analysis offer significant advantages in terms of clinical accuracy in AI-assisted dental diagnostic systems.

ROC analyses are widely acknowledged as a reliable method for assessing model robustness in medical AI applications, particularly in distinguishing challenging classes such as Grade 1 and Grade 2 [53]. The root length is calculated using the model’s estimate (PE or OD) from the before image and the root length from the after image, and the percentage of root shortening is calculated. Grades 0–3 are then assigned based on these percentages in accordance with the clinical acceptability. Therefore, ROC curves are not generated by the continuous score model; all these processes directly generate grades. ROC is obtained by dichotomising clinical classes in a one-vs-rest format. In this case, the ROC structure is multiclass, but a binary ROC is drawn for each class. The distribution in this binary structure forms the ROC curve for the relevant class. In this method, the ROC curve is generated by plotting all true sensitivity and false positive rate pairs based on the class-other-class distinction. This is the standard ROC approach used in the literature [54] for models that perform multiclass classification and do not produce continuous scores. This process is completely standard for models that do not produce softmax probabilities/continuous scores (such as random forest, SVM, decision tree, and some YOLO variants).

In this study, PE model showed a curve closer to the ideal point (0,1) than the OD model, indicating superior performance. This shows that the model provides both a high true positive rate and keeps false positives to a minimum. This situation reveals that the PE works with both high sensitivity and low false alarm rate and is more reliable in clinical decision-making processes.

The YOLOv12-based PE approach outperforms the OD model across all metrics. The PE model achieved 93% accuracy, significantly higher than the 78% observed in the OD. PE’s superior sensitivity and specificity highlight this, outperforming the OD’s. These differences are especially pronounced in Grade 1 and Grade 2 cases, where diagnostic ambiguity is typically high. The PE model’s recall and F1-score improvements in these borderline categories are important, as they directly influence the ability to detect resorption in its earliest stages, enabling timely clinical intervention.

Xu et al. [21] evaluated six CNN models for classifying OIEARR using tooth slices, achieving high AUC values. However, mild OIEARR cases were misclassified due to subtle morphological differences. In contrast, our study used a PE-based YOLOv12 model on panoramic radiographs, achieving AUC values between 0.88 and 0.95, with superior performance in early-stage resorptions (Grade 0–1). Unlike slice-based CNNs, our model used panoramic radiographs without segmentation, offering greater clinical applicability. Despite slightly lower AUCs, our approach demonstrated higher robustness and practical relevance.

Reduwan et al. [22] tested various deep learning models for automatic detection of external root resorption from CBCT data and reported that the RF + VGG combination achieved the best results with 96% AUC and 81.9% accuracy. However, this study was conducted only on CBCT scans of extracted premolars and does not sufficiently represent clinical variations.

Pirayesh et al. [55] focused on detecting canine-induced root resorption using a deep learning approach applied to CBCT images. However, their study was limited by a small sample size, a binary classification approach and dependence on CBCT imaging. These factors constrained the model’s generalisability and clinical utility. By contrast, our study used a larger, more representative dataset, applied multi-class classification to differentiate resorption severity levels and used panoramic radiographs to provide a more accessible, cost-effective solution. Notably, the YOLOv12-based PE architecture demonstrated superior sensitivity and F1 scores, particularly in the early and moderate stages of resorption. This highlights its potential for use in clinical decision support systems.

From a clinical applicability perspective, the use of YOLOv12x architectures offers a significant advantage in computational efficiency. Despite their high accuracy, these models are optimized for fast inference, and on a standard work computer, both object detection and pose estimation models can process a single dental radiograph in less than 500 ms. Therefore, the developed technique completes all computational operations and generates the final report in less than 1 s. Regarding hardware requirements, although high-performance GPUs are used during the training phase, the trained model can be easily converted to optimization frameworks such as TensorRT or OpenVINO, and is lightweight enough to run on entry-level consumer graphics cards or even modern CPUs through these techniques. No interface has been prepared for the developed technique. However, the authors aim to further develop this algorithm into a dedicated program/software in the future, and studies in this direction are currently ongoing.

Despite the promising outcomes of this study, several limitations should be acknowledged. First, the analysis was confined to the maxillary central and lateral incisors. As a result, the model’s performance in detecting resorption in premolars and molars remains untested, limiting the generalizability of the findings to other tooth groups. Second, the use of two-dimensional panoramic radiographs inherently restricts the ability to assess the depth and volumetric extent of resorptive lesions when compared to three-dimensional imaging modalities such as CBCT. Nonetheless, due to ethical considerations and concerns regarding patient safety, obtaining CBCT scans before and after treatment for every orthodontic patient is not clinically justified. Third, the study employed only the YOLOv12 architecture without comparing alternative deep learning models. Future investigations involving various architectures and hybrid frameworks with larger sample sizes may further enhance diagnostic performance and robustness.

## Conclusions

Among the two AI models evaluated, the YOLOv12-based PE model demonstrated markedly superior performance across all resorption grades, particularly in early and moderate stages where diagnostic ambiguity is highest. By leveraging fine-grained anatomical localization, the PE model achieved higher accuracy, sensitivity, and F1 scores than the OD-based approach, offering a more reliable and clinically valuable assessment framework. These findings underscore the potential of PE-based key point detection systems in enhancing diagnostic precision and enabling earlier intervention in orthodontic treatment planning. Given its accessibility, scalability, and high diagnostic accuracy, the proposed AI system represents a significant advancement in AI-assisted orthodontic diagnostics and may serve as a valuable decision support tool in routine clinical practice.

## Data Availability

The original contributions presented in the study are included in the article; further inquiries are available from the corresponding author if needed.
